# Anxiety, Depression and Quality of Life in Older Adults: Trajectories of Influence across Age

**DOI:** 10.3390/ijerph17239039

**Published:** 2020-12-04

**Authors:** Oscar Ribeiro, Laetitia Teixeira, Lia Araújo, Carmen Rodríguez-Blázquez, Amaia Calderón-Larrañaga, Maria João Forjaz

**Affiliations:** 1 Center for Health Technology and Services Research (CINTESIS), Department of Education and Psychology, University of Aveiro, 3810-193 Aveiro, Portugal; 2 Center for Health Technology and Services Research (CINTESIS), Institute of Biomedical Sciences Abel Salazar, Department of Population Studies, University of Porto, 4050-313 Porto, Portugal; lcteixeira@icbas.up.pt; 3 Center for Health Technology and Services Research (CINTESIS), School of Education, Polytechnic Institute of Viseu (ESEV.IPV), 3504-510 Viseu, Portugal; liajaraujo@esev.ipv.pt; 4 National Centre of Epidemiology, Carlos III Health Institute and CIBERNED, 28029 Madrid, Spain; crodb@isciii.es; 5 Aging Research Center, Department of Neurobiology, Care Sciences and Society, Karolinska Institutet and REDISSEC, 17165 Solna, Sweden; amaia.calderon.larranaga@ki.se; 6 National Centre of Epidemiology, Carlos III Health Institute and REDISSEC, 28029 Madrid, Spain; jforjaz@isciii.es

**Keywords:** mental health, longitudinal, SHARE, CASP-12, elderly, Portugal

## Abstract

This study focuses on the influence of anxiety and depression on individual trajectories of quality of life in old age through a longitudinal approach. A representative sample of adults aged 50+ living in Portugal and participating in wave 4 (W4) and wave 6 (W6) of the Survey of Health, Ageing and Retirement in Europe (SHARE) project was considered. Participants, 1765 at baseline (W4) and 1201 at follow up (W6), were asked about their quality of life (CASP-12) and emotional status (Euro-D scale; five items from the Beck Anxiety Inventory). Linear Mixed Effects models were performed to identify factors associated with changes in quality of life across age. Increasing age was found to have a significant negative effect on quality of life. Lower education and higher levels of depression and anxiety at baseline were also associated with worse quality of life; 42.1% of the variation of CASP-12 across age was explained by fixed and random effects, being depression followed by anxiety as the factors that presented with the highest relative importance. Both depression and anxiety play an important role in quality of life in older adults and must be acknowledged as important intervention domains to foster healthy and active aging.

## 1. Introduction

The phenomenon of demographic aging has brought such remarkable changes and implications that it is believed to become one of the most significant social transformations of the twenty-first century [1]. Countries all over the world are called to formulate policies to respond to the implications of aged societies and to guarantee that, along with good physical health, psychological well-being is assured.

In 2002, the World Health Organization presented the policy framework of “Active Aging”, which has become the leading scientific and policy conceptualization of well-being in later life [2]. Defined as “the process of optimizing opportunities for health, participation and security in order to enhance quality of life as people age” [3], this model reflects a positive and holistic vision of aging and harnesses it as both an individual aspiration and a policy goal [4].

The initial formulation of the active aging policy identified health, participation and security as the fundamental components, and more recently a fourth pillar was added: lifelong learning [4]. The ultimate goal of active aging is enjoying an old age with quality of life [3], herein understood as “individuals’ perception of their position in life in the context of the culture and value systems in which they live and in relation to their goals, expectations, standards and concerns” [5] (p. 1).

Such a positive focus towards later life, for which the Active Aging model stands, has stimulated interest in quality of life as an outcome indicator [6]. Overall, research in this field has found that older age tends to be associated with a reduction in quality of life. However, since old age includes a long period of years, it becomes crucial to know whether this reduction follows a continuous pattern or is more significant after a certain age.

Conde-Sala and colleagues [7], using data from the Survey of Health, Ageing and Retirement in Europe (SHARE), found that younger age was associated with better quality of life, with the oldest group (80 and more years) scoring lowest in quality of life. This study was cross-sectional and only analyzed individuals aged 65 and over, making it impossible to define temporal associations between age and quality of life. Netuveli and colleagues [8], with data from the English Longitudinal Study of Aging (ELSA), found an improvement in quality of life from 50 to peak at 65 years and a decline beyond 85 years. This age curve effect has led authors to distinguish between the third and fourth age in terms of quality of life, with the third age being a period of better quality of life.

Worthwhile highlighting is that, although aging is perceived to decrease quality of life, this effect may disappear when controlling for other factors. Most studies analyzing what contributes to a better quality of life in older age report the importance of health-related factors. Poor physical health, functional impairment and depression are often associated with lower quality of life [7,8,9], which explains the inclusion of health as a crucial pillar for active aging. Within health-related factors, however, the importance of mental health is too often overlooked [4], and there is a danger of policies overemphasizing physical capacity to the neglect of mental capacity [10].

Paúl, Ribeiro and Teixeira [11] have stressed the importance of differentiating among age groups when approaching the WHO Active Aging model, paying particular attention to the oldest, i.e., those aged over 75 years. Bearing this distinction in mind, they have emphasized the relevance of psychological functioning (namely the absence of psychological distress) in defining active aging, especially in the oldest age groups. Within the quality of life literature, Brown, Bowling and Flynn [12] have also brought attention to the importance of psychological well-being.

Within the mental health domain, particularly in terms of emotional status, depression has been identified as an important hindrance to quality of life in later life. Depression greatly affects daily functioning, and it has been found to have a significant impact on the psychological and social domains of quality of life [13]. Much less attention has been paid to the contribution of anxiety to quality of life, even in clinical samples [14]. This may be mostly due to the fact that the study of anxiety disorders in older adults is a relatively new field, and there is a dearth of research compared to depression and dementia. Nevertheless, anxiety was recently found to be the most prevalent mental health disorder amongst community-dwelling older adults in Europe [15], which has raised awareness of the need to further study psychosocial problems and their correlates, namely quality of life, in elderly people.

In a sample of 1680 participants over 64 years of age, Sousa and colleagues [16] found that older adults with anxiety and/or depression were more likely to report lower levels of quality of life, but studies that analyze this association are still scarce. For that reason, Hohls, König, Quirke and Hajek [17] have recently proposed a study protocol for a systematic review of the evidence on the association between anxiety, depression and quality of life in longitudinal studies, arguing that it is necessary to identify which domains of quality of life are affected by specific emotional disorders, such as anxiety, over time. This paper aims to add to the available knowledge on this matter by analyzing the influence that anxiety and depression have on individual trajectories of quality of life, and its specific domains, across old age.

## 2. Materials and Methods

### 2.1. Study Design and Setting

This is a longitudinal study including people aged 50 years or older living in Portugal that participated in waves 4 (W4) [18] and 6 (W6) [19] of the SHARE project.

### 2.2. Participants

The exclusion criteria were participants (i) who were incarcerated, hospitalized or out of the country during the entire survey period; (ii) unable to speak the country’s language; (iii) who had moved to an unknown address; (iv) with missing information on the outcome variable; and/or (v) who were diagnosed with Alzheimer’s disease, dementia or senility. The SHARE project was reviewed and approved by the Ethics Council of the Max Planck Society, and the present study was approved by the Ethics Committee of Carlos III Institute of Health (reference: CEI PI 62-2019). Additional information about the SHARE project, and more specifically about W4 and W6, can be obtained elsewhere [20,21,22].

### 2.3. Measures

The main outcome variable was quality of life as measured by the Portuguese version of the CASP-12 scale [23,24,25]. This is a shortened version of the original CASP-19 scale that was specifically used in SHARE. It is a 12-item self-assessment questionnaire that includes four domains: control, autonomy, self-realization and pleasure. Each question is rated on a four-point Likert scale (from 1 = never to 4 = often), with items 4 and 7–12 being reversely scored. The total score ranges from 12 to 48, with higher scores meaning better quality of life.

Depression was assessed by means of the Euro-D scale [26], which is also a self-assessment scale comprising 12 items (presence of depressive symptoms, pessimism, suicidality, tearfulness, guilt, sleep problems, loss of interest and appetite, irritability, fatigue, reduced concentration and loss of the capacity of enjoyment in the last month). The answer to each item is dichotomous (yes/no), and the final score ranges from 0 to 12 points, with higher scores suggesting more depressive symptoms.

For anxiety, a multi-item indicator was used, comprising five items from the Beck Anxiety Inventory [27]. This measure used in SHARE includes one item about psychological symptoms, two items about physiological symptoms, and one item about cognitive symptoms. Each item is answered on a four-point Likert scale (1 = never, 2 = hardly ever, 3 = some of the time and 4 = most of the time), with the total score ranging from 4 to 16 points; higher scores indicate higher anxiety.

Finally, socio-demographic variables (age, sex, years of education and marital status) were also considered. Education level was described according to the International Classification of Education (ISCED) [28]. A description of all variables used in SHARE W4 and W6 can be found elsewhere [18,19].

### 2.4. Statistical Analysis

A description of the sample at baseline was obtained using frequencies, means and standard deviations (SD) or medians and interquartile ranges (IQR). The psychometric properties of CASP-12 were evaluated in a previous study [23]. Linear Mixed Effects models were performed to identify potential factors associated with changes in quality of life across age. The random effects structure of the final multivariable model was chosen based on the comparison of two models: one model considering only a random intercept (individual level) and a second model considering both a random intercept (individual level) and a random slope (age level). In both models, the same fixed effects structure was used, considering all potential predictive factors at baseline. Once the random effects structure was defined, the fixed effects of the final model were selected using a backward stepwise approach. Scaled coefficients were obtained to represent comparable effects for each fixed factor. The following goodness-of-fit measures were used to compare different models: the Akaike Information Criterion (AIC), the Bayesian Information Criterion (BIC), the pseudo-R2 and the Likelihood Ratio Test (LRT). All analyses were performed in R 3.6.1 software, and a significance level of *p* = 0.05 was established.

## 3. Results

### 3.1. Sample Characteristics

The sample comprised 1765 participants at baseline (W4), of which 1201 were subsequently followed (W6). At baseline, the overall mean age was 64.7 years (SD: 9.2; range: 50–95), and 55.3% were female. The majority were married (1421, 80.5%), and the median of years of education was 4.0 years (IQR: 5.0 years). The mean depression score was 3.32 (SD: 2.53), and the mean anxiety score was 9.32 (SD: 3.26). Information on the baseline sample characteristics can be found in Table 1.

### 3.2. Changes in Quality of Life across Age

**Figure 1:**
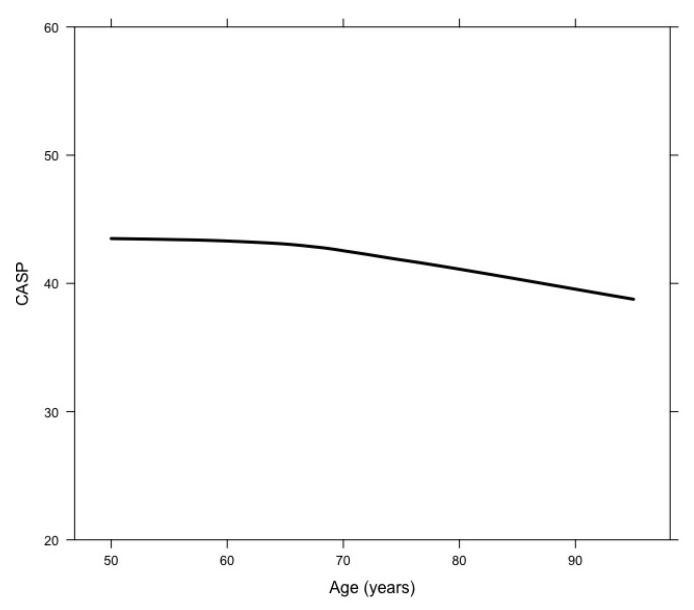
Mean CASP-12 trajectory across age.

Figure 1 shows changes in CASP-12 across age. The mean score of CASP-12 decreased with age, which was confirmed by results from the empty model (B = −0.07; standard error = 0.01; *p* < 0.001).

**Figure 2:**
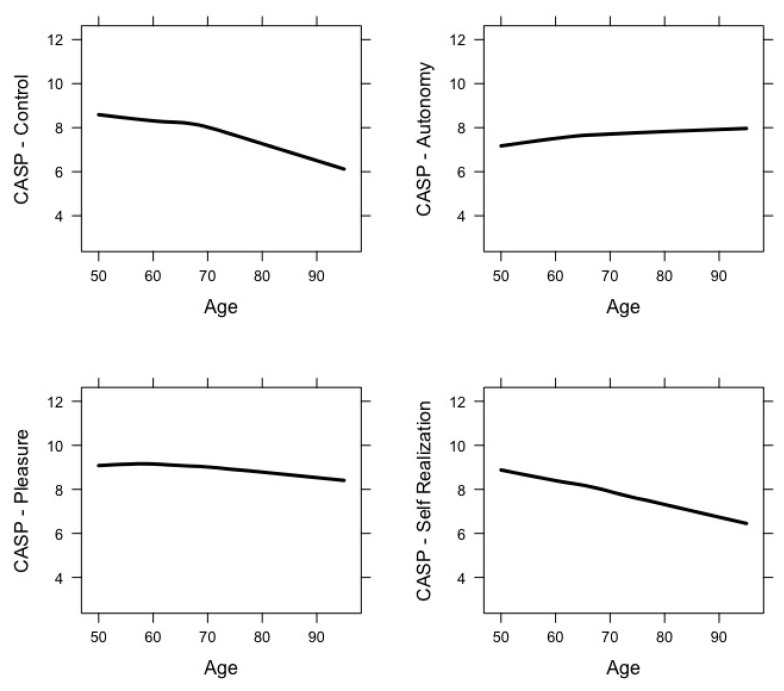
Domain-specific mean CASP-12 trajectories across age.

Figure 2 shows changes in the four domains of CASP-12 across age. The mean scores for the Control and Self-Realization domains decreased with age; the Pleasure domain remained constant across age with a slight decrease in more advanced ages, whereas the Autonomy domain slightly increased over time.

Following the modelling strategy described in the Methods section, the final set of predictors of quality of life are shown in Table 2. Model 1 comprised all fixed and random effects considered (full model). Model 2 was similar but excluded the random slope effect. Comparing the AIC and BIC of both models and based on the LRT (Chi-Squared = 17.95, df = 1, *p* < 0.001), the inclusion of a random slope was warranted since it significantly improved model fit. Model 3 was similar to Model 1, but excluded marital status as a fixed effect factor. When comparing these two models based on the LRT (Chi-Squared = 4.057, df = 3, *p* = 0.255), no differences were found, suggesting that marital status was not significantly associated with changes in CASP-12 across age. Additionally, the AIC and BIC were slightly smaller for Model 3 compared with Model 1, indicating that the former was better fit.

Based on results from Model 3, increasing age (as the time-dependent factor) was significantly associated with declines in quality of life. Additionally, more years of education and lower levels of depression and anxiety at baseline were significantly associated with better quality of life. Depression presented the highest relative importance out of the set of fixed factors, followed by anxiety.

Model 3 showed a pseudo-R^2^ equal to 0.421, i.e., 42.1% of the variation of CASP-12 across age could be explained by the fixed and random effects considered in the model.

## 4. Discussion

Findings from this study stress that, overall, quality of life diminishes with age. It also acknowledges that the effect of age is independent of other sociodemographic characteristics (i.e., sex and years of education) and emotional status. The study also shows that lower education and higher levels of depression and anxiety are significantly associated with worse future quality of life, depression being the factor with highest relative importance, followed by anxiety.

Previous research has largely demonstrated that quality of life tends to diminish with age, which is consistent with our findings. Nonetheless, such a change may not be linear, as several studies have reported that it tends to continue to increase through early old age before decreasing in later older age (e.g., [29,30]). This was, however, not the case in our study, where a small but constant drop was observed from approximately age 70 onwards. Although the importance of age itself has been highlighted in several studies (e.g., [31]), the association between age and quality of life is intricate, as several studies show that quality of life and its domains vary between individuals and groups (e.g., [32]), and that a wide diversity of associated variables must be taken into consideration (e.g., [30]). In our study, the observed constant drop in the overall score of quality of life may be associated with the instrument used here for assessing quality of life. When taking a closer look at the observed changes in the four domains of CASP-12 across age, two were found to clearly decrease with age (Control and Self-realization), whereas the Pleasure domain remained mostly constant with a slight decrease in very old ages, and the Autonomy domain somewhat increased. Previous data on the psychometric properties of the Portuguese version of this instrument have shown some inadequacies in terms of acceptability, internal consistency, and structural and construct validity, precisely in the Autonomy and Pleasure domains [23], echoing preceding concerns regarding the scale’s factorial structure and even the potential need for a revised shorter version of the scale [33]. As the use of the total score is preferred over individual domain scores, only cautious interpretations can be made as to whether and which specific quality of life domains are particularly affected over time. Bearing this important limitation in mind, what this study suggests is that one’s ability to be self-determined and the absence of unwanted interference from others (Autonomy domain) may not be as compromised across age as the other CASP-12 domains. Nevertheless, the aforementioned psychometric limitations of the scale lead us not to overvalue the age-related trends observed for the Autonomy domain.

Our second major finding confirms the well-established influence of depressive symptoms on quality of life. The important role played by anxiety reinforces the need to pay further attention to this condition. A recent large-scale study from SHARE showed that increases in anxiety in late adulthood were linked with age-associated losses in physical and cognitive functioning [34], highlighting that aging is in fact accompanied by losses in several health domains that certainly demand increased coping efforts. Considering that previous research has confirmed the impact of anxiety on quality of life [35], increasing the effort to better understand and prevent anxiety in later life, either at a clinical or subclinical level, is a vital goal for achieving active and healthier aging.

Worth noting are the cultural specificities linked to the Portuguese origin of study participants, in terms of the prevalence and distribution of emotional disorders in later life. A recent study based on a representative cohort of 10,661 Portuguese older adults revealed an estimated prevalence of anxiety and depression of 9.6% and 11.8%, respectively, such cases being associated with a higher likelihood of presenting with lower levels of quality of life [16]. Although these figures are presented by the authors as being considerably lower than those found in other international studies from Australia, the Americas and even Europe, the differences are most likely due to disparities among populations, age group cutoffs and assessment tools. All in all, there is a need to prevent psychological distress and emotional illness in the Portuguese older population, and to acknowledge the potential consequences both anxiety and depression may have on well-being and quality of life in the long run. Moreover, several Portuguese studies have highlighted the existence of specific subgroups to whom particular attention must be paid, namely older adults with severe cognitive impairment and with high levels of functional dependency [32].

Regarding the impact of educational level, a previous study on factors influencing quality of life in people aged 65 years and over in Europe revealed that higher education was only associated with better quality of life in Eastern European and Mediterranean countries, such as Portugal [7]. This is in line with our findings as well as with those obtained by Henriques and colleagues [36], who recently emphasized the importance of education and social support for Portuguese older adults’ quality of life. These findings highlight the impact that education has on quality of life and underline how this construct relates to welfare regimes [37], particularly concerning societies’ socioeconomic conditions.

A final word must be added on the absence of sex differences found in this study and the fact that marital status was not a significant predictor of CASP-12 changes across age. Studies have found contradictory results in regard to sex, with some finding worse quality of life in men (e.g., [8]) and others in women (e.g., [7]). Nevertheless, in a study by Cantarero-Prieto et al. [38], the effect of gender found across European counties was lost when only Southern European countries were considered. As for marital status, despite being an objective and sociodemographic variable with a relatively small influence, it is considered to be an important determinant of quality of life [12]. The lack of association for these factors in our study may be related to social and cultural aspects, which should be further researched.

This study has some limitations which should be acknowledged. First, assuming a linear association between CASP-12 and age might not be optimal, since other studies have revealed non-linear associations. Second, as the focus of this paper study was on mental health, we did not consider other covariates such as functional status and physical conditions that could influence the relation between CASP-12 and age. Third, few time points were included in the present study, but further work is already being undertaken to obtain longer follow-ups. Despite its limitations, this study provides longitudinal information about a central aspect of older peoples’ well-being, that is, the impact of mental health on their quality of life.

## 5. Conclusions

This study showed that quality of life decreases with age, independently of sex, education and emotional mental health conditions. Moreover, depression and anxiety decisively shape individuals’ trajectories of quality of life over time. Portugal, like most other countries, will be aging rapidly in the coming years; about 47.1% of the total population will be over 55 years by 2050 [39]. Considering such a scenario, optimizing the aging process and the quality of life of older adults is an important goal. Active aging interventions must necessarily incorporate the mental health dimension in order to minimize the prevalence of psychological distress, emotional illnesses and their respective consequences. Specifically, screening and treating for depression and anxiety symptoms among older adults might result in a better quality of life, both in the short and long run.

## Figures and Tables

**Table 1:** Baseline sample characteristics.

	*n* (%), Mean (SD) or Median (IQR)
Sex, *n* (%)	
Female	976 (55.3)
Male	789 (44.7)
Age, mean (SD)	64.7 (9.2)
Years of education, median (IQR)	4.0 (5.0)
Marital status, *n* (%)	
Never married	63 (3.6)
Married	1421 (80.5)
Divorced	79 (4.5)
Widowed	202 (11.4)
Depression, mean (SD)	3.32 (2.53)
Anxiety, mean (SD)	9.32 (3.26)

Depression measured though the Euro-D scale; range from 0 to 12 points. Anxiety assessed through the Beck Anxiety Inventory; range from 4 to 16.

**Table 2:** Linear Mixed Effects models with predictors of CASP-12.

	Model 1			Model 2			Model 3		
***Fixed Effects***									
	**Scaled B-Coefficient (SE)**	**95% CI**	***p***	**Scaled B-Coefficient (SE)**	**95% CI**	***p***	**Scaled B-Coefficient (SE)**	**95% CI**	***p***
Intercept	32.1 (0.50)	31.1–33.0	<0.001	32.1 (0.50)	31.1–33.1	<0.001	32.6 (0.13)	32.4–32.9	<0.001
Age	−0.30 (0.10)	−0.50–0.10	0.004	−0.32 (0.10)	−0.53–−0.15	0.001	−0.32 (0.10)	−0.51–−0.13	0.001
Sex, male [ref. female]	−0.02 (0.20)	−0.41–0.37	0.914	−0.02 (0.20)	−0.42–0.36	0.928	−0.04 (0.20)	−0.35–0.42	0.855
Years of education	0.68 (0.10)	0.49–0.87	<0.001	0.68 (0.10)	0.49–0.88	<0.001	0.67 (0.10)	0.47–0.86	<0.001
Marital status [ref. never married]									
Married	0.67 (0.51)	−0.33–1.66	0.188	0.55 (0.51)	−0.45–1.53	0.274			
Divorced	0.03 (0.66)	−1.26–1.32	0.966	−0.06 (0.66)	−1.33–1.24	0.931			
Widowed	0.38 (0.59)	−0.77–1.53	0.515	0.34 (0.58)	−0.77–1.51	0.557			
Depression	−1.90 (0.11)	−2.11–−1.69	<0.001	−1.89 (0.11)	−2.14–−1.71	<0.001	−1.91 (0.11)	−2.12–−1.70	<0.001
Anxiety	−0.82 (0.11)	−1.03–−0.60	<0.001	−0.83 (0.11)	−1.02–−0.60	<0.001	−0.81 (0.11)	−1.02–−0.59	<0.001
***Random effects***									
σ (intercept)	2.053			2.260			2.066		
σ (age)	1.012						0.974		
σ (residual)	4.016			4.034			4.019		
***Goodness-of-fit***									
AIC	17453.4			17458.5			17451.7		
BIC	17525.3			17524.4			17505.7		
Pseudo-R^2^	0.421			0.437			0.421		
